# P260: Step assessment of nosocomial infections control in Sengal (Pronalin)

**DOI:** 10.1186/2047-2994-2-S1-P260

**Published:** 2013-06-20

**Authors:** BK Oumar Thiam

**Affiliations:** 1 Coordinator of PRONALIN, Dakar, Senegal

## Introduction

As a member state of WHO, Senegal, has committed to take up the global challenge of nosocomial infections control by setting up a national program since 2004. A road map including administrative and organizational measures as well as technical activities had been shared with all health structures.

## Objectives

The objective of this work is to evaluate the implementation of the program.

## Methods

A supervision guide had been elaborated based on the activities outlined in the roadmap. We conducted a formal evaluation of these activities.

## Results

95% of the health structures have set up an institutional framework as well as prioritized workgroups; hand washing activities and biomedical waste management has been carried out partially. 95% of health structures did not carry out any activity on care infection surveillance; only 38% of the health institutions have set up a local section for patient security.

## Conclusion

Setting up an institutional framework as well as prioritized workgroups was a success. However, it should be mentioned that implementation of the roadmap activities could have been better.

## Competing interests

None declared

